# Unhealthy eating practices of city-dwelling Africans in deprived neighbourhoods: Evidence for policy action from Ghana and Kenya

**DOI:** 10.1016/j.gfs.2020.100452

**Published:** 2020-09

**Authors:** Michelle Holdsworth, Rebecca Pradeilles, Akua Tandoh, Mark Green, Milkah Wanjohi, Francis Zotor, Gershim Asiki, Senam Klomegah, Zakia Abdul-Haq, Hibbah Osei-Kwasi, Robert Akparibo, Nicolas Bricas, Carol Auma, Paula Griffiths, Amos Laar

**Affiliations:** a French National Research Institute for Sustainable Development (IRD), NUTRIPASS Unit: IRD-Univ Montpellier-SupAgro, Montpellier, France; b School of Sport, Exercise and Health Sciences, Loughborough University, UK; c School of Public Health, University of Ghana, Accra, Ghana; d School of Environmental Sciences, University of Liverpool, Liverpool, UK; e African Population and Health Research Center (APHRC), Nairobi, Kenya; f University of Health and Allied Sciences, Ho, Ghana; g School of Health and Related Research, University of Sheffield, Sheffield, UK; h Department of Geography, University of Sheffield, Sheffield, UK; i French Agricultural Research Centre for International Development (CIRAD), Montpellier, France

**Keywords:** Eating practices, Unhealthy foods, Food environment, Africa, Ghana, Kenya, Cities

## Abstract

Growing urbanisation in Africa is accompanied by rapid changes in food environments, with potential shifts towards unhealthy food/beverage consumption, including in socio-economically disadvantaged populations. This study investigated how unhealthy food and beverages are embedded in everyday life in deprived areas of two African countries, to identify levers for context relevant policy. Deprived neighbourhoods (Ghana: 2 cities, Kenya: 1 city) were investigated (total = 459 female/male, adolescents/adults aged ≥13 y). A qualitative 24hr dietary recall was used to assess the healthiness of food/beverages in relation to eating practices: time of day and frequency of eating episodes (*periodicity*), length of eating episodes (*tempo*), and who people eat with and where (*synchronisation*). Five measures of the healthiness of food/beverages in relation to promoting a nutrient-rich diet were developed: i. nutrients (energy-dense and nutrient-poor -EDNP/energy-dense and nutrient-rich -EDNR); and ii. unhealthy food types (fried foods, sweet foods, sugar sweetened beverages (SSBs). A structured meal pattern of three main meals a day with limited snacking was evident. There was widespread consumption of unhealthy food/beverages. SSBs were consumed at three-quarters of eating episodes in Kenya (78.5%) and over a third in Ghana (36.2%), with those in Kenya coming primarily from sweet tea/coffee. Consumption of sweet foods peaked at breakfast in both countries. When snacking occurred (more common in Kenya), it was in the afternoon and tended to be accompanied by a SSB. In both countries, fried food was an integral part of all mealtimes, particularly common with the evening meal in Kenya. This includes consumption of nutrient-rich traditional foods/dishes (associated with cultural heritage) that were also energy-dense: (>84% consumed EDNR foods in both countries). The lowest socio-economic groups were more likely to consume unhealthy foods/beverages. Most eating episodes were <30 min (87.1% Ghana; 72.4% Kenya). Families and the home environment were important: >77% of eating episodes were consumed at home and >46% with family, which tended to be energy dense. Eating alone was also common as >42% of eating episodes were taken alone. In these deprived settings, policy action to encourage nutrient-rich diets has the potential to prevent multiple forms of malnutrition, but action is required across several sectors: enhancing financial and physical access to healthier foods that are convenient (can be eaten quickly/alone) through, for example, subsidies and incentives/training for local food vendors. Actions to limit access to unhealthy foods through, for example, fiscal and advertising policies to dis-incentivise unhealthy food consumption and SSBs, especially in Ghana. Introducing or adapting food-based dietary guidelines to incorporate advice on reducing sugar and fat at mealtimes could be accompanied by cooking skills interventions focussing on reducing frying/oil used when preparing meals, including ‘traditional’ dishes and reducing the sugar content of breakfast.

## Highlights

• Growing urbanisation in African countries, such as Ghana and Kenya is accompanied by rapid changes in food environments.
• Investigating unhealthy food practices in deprived areas of African cities identified levers for contextualised policy.
• Widespread consumption of unhealthy food/beverages including energy dense nutrient rich foods associated with tradition.
• A structured three main meals a day pattern with limited snacking was evident, with eating with family or alone common.
• Inter-sectoral policy promoting nutrient-rich diets to prevent all forms of malnutrition in deprived urban areas is needed.

## 1 Introduction

Africa is experiencing a nutrition transition with changing dietary habits and food environments related to urbanisation (Imamura et al., 2015; Agyemang et al., 2016, Holdsworth and Landais, 2019), accompanied by rising obesity and diet-related non-communicable diseases (DR-NCDs) (Naghavi et al., 2017) and persistent micronutrient deficiencies/undernutrition, affecting all socioeconomic groups. Nutrition transition is characterized by increased consumption of added sugar, fat (particularly oils), animal-source foods and decreased consumption of coarse grains, staple cereals and pulses (Popkin and Gordon-Larsen, 2004; Hawkes et al., 2017). Ghana and Kenya typify this dietary and epidemiological transition (Ghana DHS, 2015; Kenya DHS, 2015; Rischke et al., 2015; Agyemang et al., 2016; Cira et al., 2016; Ofori-Asenso et al., 2016; 2017; Rousham et al., 2020), which they have recognised as a pressing public health concern through the development of national policies to prevent NCDs, incorporating interventions and policies to promote healthier diets (Ministry of Health Ghana, 2012; Ministry of Health Kenya, 2015). However, the evidence for developing policy action within the African region comes mainly from high income countries and is not tailored for low socioeconomic groups within African cities. Therefore, it is important to investigate the eating practices that people develop in changing urban food environments, so that context and culturally-sensitive policies and interventions can be developed; this is difficult to achieve without locally relevant evidence.

The overconsumption of unhealthy diets with a high concentration of calories and low micronutrients (energy-dense and nutrient-poor -EDNP), is implicated in the onset of multiple forms of malnutrition (Pradeilles et al., 2019-a). Eating practices may also evolve in response to changing contexts (Jastran et al., 2009), such as that accompanying the nutrition transition. Eating practices are shaped by many social, material, economic and cultural factors (Warde et al., 2007; Jastran et al., 2009; Southerton et al., 2011; Warde 2015; Osei-Kwasi et al., 2020) in people's food environments and are *‘closely tied to the routines and rhythms of everyday life'* (Horton et al., 2017). Policies and interventions to promote healthier food consumption may be more effective when they address the dynamics of eating practices, which requires an exploration of how food consumption is structured and organised in social practices (Shove et al., 2012), such as the time of day and frequency of eating episodes (*periodicity*), length of eating episodes (*tempo*), and who participants eat with and where (*synchronisation*). The *periodicity* with which people eat may have negative effects on health, for example eating more frequent and irregular meals can have a detrimental impact on body weight (St-Onge et al., 2017). *Periodicity* also includes consumption of specific foods at certain times of the day that mark the passing of periods of time (breakfast–lunch–dinner) (Southerton et al., 2011). *Tempo* is integral to different types of eating episode, which may, for example, be relatively fast when eating alone compared with when eating with others (Southerton 2011). *Synchronisation* requires the co-ordination of people and practices. Eating practices are usually synchronised with other practices such as work routines and social lives (Southerton 2011). Hence, by investigating eating practices in deprived communities in urban Africa, emerging public policies and recommended interventions can be developed that are sensitive to the context and therefore more likely to lead to healthier food environments and the consumption of healthier diets.

There is a lack of evidence about how unhealthy food and beverage consumption is embedded in everyday life in African cities, including for the urban poor (Osei-Kwasi et al., 2020). Therefore, the objective of this study was to ascertain how unhealthy food and beverage consumption is embedded in everyday life in deprived areas of two African countries, to identify levers for context relevant policy.

## 2 Methods

### 2.1 Ethical approval

Ethical approval for the study was acquired by each institution involved in the data collection process. In Ghana, ethical approval was obtained from the Ghana Health Service Ethics Review Committee (references: GHS-ERC 07/09/16 and GHS-ERC 02/05/17). In Kenya, ethical approval was obtained from Amref Health Africa (reference: ESRC P365/2017). The University of Sheffield and Loughborough University recognised both of these ethical review boards as meeting their ethical standards. Additional ethical approval was obtained from the University of Liverpool (references: 1434 and 2288) and Loughborough University (reference: R17 -P142). Written informed consent was obtained from adults and assent from legal guardians of participants <18 y.

### 2.2 Selecting neighbourhoods and participants

Comparable studies were conducted with adolescents/adults aged ≥13 y (male and female) in deprived neighbourhoods of three rapidly growing cities in Ghana (Accra and Ho) and Kenya (Nairobi). In Ghana, James Town was selected from a list of poverty endemic areas in Accra (CHF International, 2010) and Dome was selected from a list of poor areas in Ho (UN-HABITAT, 2009). In Nairobi, Makadara was selected amongst high deprivation areas (Kenya National Bureau of Statistics et al., 2015) that were judged to be safe to work in by the research team. To select participants in these low income neighbourhoods, quota sampling was used to gain a broad sample based on age, body mass index (BMI), occupation and socio-economic status (SES). These socio-demographic factors were included to ensure a diverse sample. The target sample was: n = 294 Ghana (192 in Accra and 96 in Ho); n = 144 Kenya). These quota sampling frames differ because the data from Ghana combine two separate sister projects (DFC and TACLED) with different target populations. The DFC project was only conducted in Ghana (Accra and Ho) whilst the TACLED project was conducted in both Ghana (Accra only) and Kenya (Nairobi). (**Supplementary file 1: Quota sampling**).

To classify participant's SES for quota sampling, we applied different methods in Ghana and Kenya. In Ghana, we derived household SES scores from 13 questions used in the Ghana EquityTool. Household scores were then compared to the average scores for urban Ghana and SES quintiles were subsequently derived. Participants were further classified into three groups: ‘lowest SES’ (1st quintile); ‘low to middle SES’ (2nd and 3rd quintiles) and ‘high SES’ (4th and 5th quintiles). For this study, only participants in the 1st and 2nd groups, representing the ‘lowest SES’ and ‘low to middle SES’ were selected. In Kenya, participants' SES was derived from their total household expenditure; those spending <23,674Ksh/month were classified as lowest SES while those spending 23,674Ksh to 199,999Ksh/month were classified as low-middle SES based on the Kenya National bureau of Statistics classification (2015).

### 2.3 Assessing food intake

In both countries, an interviewer-led questionnaire was administered using electronic data capture (CsPro version 6.3/Survey CTO version 2 on a Samsung Galaxy tab-4) to obtain information relating to socio-demographic characteristics and 24hr food consumption and eating practices. For the latter, interviewers noted all food and drink consumed by participants in/out-side of the home in the previous 24hr. They also recorded how long an eating episode lasts (‘tempo’), time of day of the eating episode (‘periodicity’), who participants eat with and where (‘synchronisation’) (**Supplementary file 2: 24hr recall**). To facilitate data collection, a pre-defined list of food items (Ghana *n* = 229 and Kenya *n* = 270) was inputted into the electronic data collection template. The development of these food lists was informed first from an earlier study in Ghana (Osei-Kwasi et al., 2019-a) and adapted for Kenya, the subsequent food list was discussed with local partners in all cities and communities to incorporate their knowledge of food consumption locally. Data were collected between June-December 2017, so over a 7 month period covering both dry and rainy seasons, therefore seasonality did not impact on food consumption data. From local knowledge, the day to day variation of dietary intake is low in the context of these urban poor communities, meaning that one 24hr recall is probably a good reflection of eating practices; we also asked participants whether this was a usual day before beginning the 24 h recall and arranged to return on another day if it was not.

#### 2.3.1 Categorising foods and beverages

Foods that were consumed were categorised into 26 food groups to explore how healthy they were and by comparing with those expected for countries undergoing nutrition transition (Hawkes et al., 2017; Rousham et al., 2020) (Table 1).

**Table 1:** Food and beverage items consumed per country.

	Food group	Food item: Kenya	Food items: Ghana
1	Fats and oils (oils, spreading fats and fats)	Margarine, butter, peanut butter, vegetable oil, corn oil, kimbo/kasuku/cowboy/chipsy (vegetable fats)	Palm oil, margarine, coconut oil
2	Sugar and sweet spreads	Jam, sugar, sugarcane, honey, sukari nguru (molasses)	Sugar, other sugar and sweet spreads
3	Red meat, poultry, offals & giblets	Beef, pork, minced meat, liver, goat, matumbo (fried cow/goat intestines), fried chicken	Pork, fried chicken, boiled chicken, grilled chicken, turkey, goat, beef, grilled beef, fried beef, wele (cow hide or/feet), liver and giblets, offal, guinea fowl, duck
4	Fish and shellfish	Fish non-fried/fish fried. *NB. fish consumption was very low in Kenya so different types were not identified*	Fish non-fried (barracuda, tuna, tilapia, salmon, cassava fish, mudfish, sardine, kpanla/adziador (Marine-sourced fish, usually smoked), fish fried (tilapia fried, tuna fried, kyenam (fried fish), seafood/shellfish (snail, clams,/adodi, crab, oysters, octopus), dried fish (anchovies), canned fish, smoked fish, kako (salted fish)
5	Eggs	Scrambled egg, poached egg, fried egg, boiled egg, omelette	Scrambled egg, fried egg, boiled egg
6	Processed meat	Smokies (precooked smoked sausage), mutura (African sausage)	Fried sausage, corned beef
7	Dairy products	Milk, sweetened condensed milk, unsweetened condensed milk, soya milk, coconut milk or cream fermented milk (maziwa mala (fermented milk), mursik (fermented milk flavoured with charcoal)	Sweetened condensed milk, powdered milk, evaporated milk, milk, flavoured yoghurt, burkina drink (ground millet/maize and pasteurized milk)
8	Sweetened tea & coffee	Sweetened tea, sweetened coffee	Sweetened tea, sweetened coffee
9	Sugar Sweetened Beverages (except tea/coffee)	Non-alcoholic beer, sodas, fruit based drinks, squashes, cocoa milk drink (Milo etc)	Light and soft drinks, sodas and sweetened beverages, fruit based drinks, cocoa milk drink (Milo, chocolim, richoco), Sobolo (hibiscus tea: dried hibiscus leaves and sweetened with sugar)
10	Alcoholic beverages	Beer, wine, spirit	Beer, wine
11	Cakes and sweets	Doughnut, mandazi (African doughnut- deep fried), scone, cake, biscuits cookies, chocolate, sweets and toffee, mabuyu (sweetened/flavoured baobab seed), ngumu (hard cake), pancake	Sweet pie or tart, pastries, biscuits(imported/local), chocolate, sweets and toffee, ice cream, groundnut cake, doughnuts, bofrot (dry doughnuts)
12	Crisps and crackers	Crisps, chips (snack made from flour dough fried)	Plantain crisps, chips (snack made from bread flour dough fried)
13	Modern mixed dishes	No consumption	Fried rice, fried noodles
14	Traditional mixed dishes	Githeri (maize & beans), muthokoi (dehusked maize & beans), mukimo (potatoes, vegetable, pumpkin leaves, maize and beans), pilau (rice, vegetables, spices & meat), meat stew, fish stew, vegetable stew	Bean stew, eto (boiled plantain or yam with palm oil), waakye (cooked rice and beans meal), red (fried plantain with bean stew), jollof rice, egg stew, garden egg stew, cabbage stew, tomato sauce and stew, okro stew, nkontomire stew (local spinach stew), moringa stew (made with moringa oleifera leaves)
15	Condiments	Tomato and chilli sauce (ketchup), dried chilli, tomato paste	Shito (a traditional condiment/very hot sauce), pepper sauce
16	Wholegrain cereals	Whole (brown) bread, local brown rice, whole meal (brown) chapatti, whole meal ugali (whole corn flour meal), whole meal porridge, boiled maize, roasted maize	Local brown rice, boiled corn meal, maize sorghum, whole grain bread (seeded), whole (brown) bread, maize (boiled, roasted), millet porridge, other wholegrain cereals
17	Refined cereals	White bread, white rice, noodles, macaroni, white chapatti, white ugali (dehusked corn flour meal), white naan, refined porridge	White bread (sugar bread, butter bread, tea bread), white crisp bread, oats, white rice, pasta, macaroni, hot cereals/porridge/maize porridge/rice porridge, tapioca, tombrown (porridge of roasted corn/cereal flour), indomie/noodles, hausa koko (spicy millet porridge)
18	Roots/tubers not fried	Bananas (roasted/boiled), arrowroots, potatoes (roasted/boiled), sweet potatoes (roasted/boiled), yam	Plantain (roasted/boiled), cassava (boiled), gaari/gari (cassava powder), yam, fufu (boiled cassava, yam, plantain or cocoyam), Konkonte (fufu made solely from cassava flour/water)
19	Roots/tubers fried	Fried potatoes, fried sweet potatoes, fried arrowroots, fried bananas, fried bhajia	Plantain fried, sweet potatoes fried, yam fried
20	Legumes and pulses	Beans, lentils, ndengu (green grams), mbaazi (pigeon peas), njahi (black beans)	Baked beans, red beans
21	Nuts and seeds	Groundnuts	Agushi (melon seeds), groundnuts
22	Fruit	Orange, watermelon, ripe pawpaw, pineapple, apple, passion fruit, banana, lemon, avocado	Aluguntungui (sour soup), banana, watermelon, avocado, orange, pineapple, pear, mango, coconut, fruit juices (unsweetened), Pawpaw
23	Vegetables	Osuga/sucha/managu (African nightshades), cucumber, peppers, pumpkin, tomatoes, red or yellow pepper, green peas, green beans, carrots, kales, spinach, eggplant, mushrooms, onions, chicory, sukuma wiki (kale), kanzira (Ethiopian kale), saga (spider plant), mrenda (Jute mallow), mitoo (Bush Okra), garlic, kunde (cow pea leaves), terere (amaranth)	Green leaves, spinach, lettuce, chinese and white Cabbage, tomatoes, peppers, carrots, cucumber, eggplant, green beans, onions and garlic, mushrooms, pumpkin, bottle gourd, okro, turkey berries, other locally available leaves and traditional vegetables
24	Savoury pies	Vegetable samosa, meat samosa,	Meat pie, fish pie, koose (bean cake; spicy black-eyed pea fritter)
25	Fermented and non-fermented grain products	No consumption	Akple (unfermented cereal meal), T.Z/Tuo Zaafi (unfermented cereal meal), kenkey-Ga/Fante (fermented cereal meal), banku (fermented cereal meal), aboloo (fermented cereal meal), mashed kenkey (kenkey with sugar, milk and possibly peanut)
26	Soups	Tomato soup, vegetable soup, bone soup	Ademe soup (made from leaves of jute plant), light soup, vegetable soup, agushie soup (melon seeds), amma soup (green leafy vegetable), groundnut soup, lentil pea and bean soup, okro soup, palmnut soup, nkontomire soup (made from local spinach leaves), other soup

Table 1. List of all foods and beverages consumed.

Five measures of healthiness were used to classify foods (Table 2) in terms of: i. Their nutrient/energy density; and ii. Based on the unhealthy types of foods/beverages to prevent DR-NCDs (sweet foods, sugar sweetened beverages (SSBs) and fried foods). SSBs were defined to include cold and hot drinks with added sugar as well as non-diet soft drinks, regular soda, iced tea, sports drinks, energy drinks, fruit punches, sweetened waters following standard definitions (von Philipsborn et al., 2019). There is overlap between categories i and ii, but they serve different purposes. Whilst energy and nutrient density of foods provides a technically correct classification, it does not tell us about the unhealthy food groupings, which is particularly useful for communicating public health interventions, such as developing food-based dietary guidelines (FBDGs). Therefore, classification into unhealthy food types (Table 2) was undertaken by categorising individual food items into these types, based on cooking method and high total fat/sugar content.

**Table 2:** Classification of foods and beverages into unhealthy categories.

	Kenya (Nairobi)	Ghana (Accra and Ho)
**Classification based on nutrient and energy density**		
EDNP (energy dense, nutrient-poor foods)***Energy Dense*** *(>*225 kcals*/100g)****Nutrient Poor*** *(<10% for nutrient rich index score)*	Matumbo, mutura honey, jam, sweets and toffee, sugar, cake, scone, biscuit cookies, white chapatti, doughnut, margarine, butter vegetable fats/oils fried bhajia, sukari nguru	Fried red meat (beef, goat, pork, bush meat, cat meat), fried chicken, duck, bofrot, meat pie, fried sausage, TZ, sugar, sweet spreads, biscuits, sweets and toffee, doughnuts, tapioca, vegetable oil, margarine
EDNR (energy dense, nutrient-rich foods)***Energy Dense*** *(>*225 kcals*/100g)****Nutrient Rich*** *(≥10% for nutrient rich index score)*	Pancake, crisps, mabuyu, mandazi, ngumu, vegetable/meat samosa, roasted maize, local brown rice, wholemeal chapati, bread, fried chicken, pork, smokies, peanut butter, groundnuts, unsweetened condensed milk	Bread, yam, plantain, maize, burkina drink, powdered milk, gari, konkonte, waakye, koose, boiled red meat (beef, goat, pork, bush meat, cat meat), corned beef, tilapia fried, octopus, groundnuts
**Classification based on food types**		
Fried foods (fried through cooking process)	Fried chicken, fried egg, fried sausage, koose, fried octopus, fried plantain/banana, fried sweet potato/potato, fried tilapia, fried yam, fried arrowroots, chips (flour dough fried), vegetable/meat samosa, crisps, fried bhajia	Fried chicken, fried egg, fried sausage, koose, fried octopus, fried plantain, fried sweet potato, fried tilapia, fried yam
Sweet foods (added sugars)	Sweets/toffee, chocolate, sugar, sugarcane, jam, honey, mandazi, doughnut, ngumu, scone, biscuit/cookies, sukari nguru, sugar cane juice, cake	Sweets/toffee, chocolate, bofrot, sugar, sweet pie/tart, tombrown, sugar/sweets
Sugar Sweetened Beverages (SSBs)	Sweetened tea/coffee, sodas sweetened fruit juices, squash, fruit based drink	Sweetened tea/coffee, burkina drink, sobolo, sodas and sweetened beverages

Full definitions of Ghanaian and Kenyan dishes are in Table 1.

Table 2. Classification of foods and beverages into food groups.

Combining the nutrient and energy density information of each food/beverage allowed us to classify food items as EDNP (represents ‘unhealthy’ foods/beverages). We were also interested in consumption of energy-dense, nutrient-rich (EDNR) foods/beverages because of their potential contribution to obesity and DR-NCDs, but also their importance in providing micronutrients in the context of multiple burdens of malnutrition in Ghana and Kenya.

Food items were classified as energy dense if >225 kcal/100 g (WCRF/AICR, 2007). We classified foods based on their nutrient composition by assigning each food with a nutrient density score to reflect its nutrient quality based on previously validated approaches (e.g. Drewnowski, 2005; 2010; Drewnowski and Fulgoni, 2014). The score incorporated 11 nutrients to encourage (protein, fibre, vitamins A, C, E and iron, calcium, potassium and magnesium, folate and zinc) and three nutrients to limit (total fat, total sugars, sodium) based on balancing the public health nutrition context with the availability of food composition data for the selected nutrients in Ghana (Abdul-Haq et al., 2018) and in Kenya. For each food item consumed, nutritional information per 100 kcal for the 11 nutrients and energy density were extracted from a combination of food composition tables based on their rigour and local relevance. Nutritional content (both macro- and micro-nutrient information) for each of these unique food items was then identified using a combination of food composition tables) (6 for Ghana and 4 for Kenya). The primary tables used were: The West African Food Composition Table (Ghana) and the Kenyan Food Composition Table (**Supplementary file 3: Food composition tables and nutrient profiling method**). Where food composition data were unavailable for nutrients and/or energy density in any of these tables (38 foods in Ghana; 2 in Kenya), they were substituted with similar food items agreed by co-authors (AT, SK, MG, MH, MW, NB, RP). Using USDA dietary recommendations, the % daily value of all nutrients was calculated per 100 kcal. Nutrient density scores were generated by subtracting the sum of the nutrients to limit from the sum of the positive nutrients to encourage. Each food item was categorised as nutrient dense if the nutrient density score was ≥10% and nutrient poor if <10% applying widely used cut-offs (U.S. Department of Health and Human Services, 2013).

### 2.4 Assessing eating practices: periodicity, tempo and synchronisation

As an integral part of the 24hr recall (Supplementary file 2: 24hr recall), questions were asked to assess routines of eating practices in relation to the time of day and frequency of eating episodes (periodicity), length of eating episodes (tempo), and who people eat with and where (synchronisation). An eating episodes was defined as any eating occasion that involves consumption of any food/beverage (except water alone) by participants. We chose this term because it reflects the lived experiences of individuals as part of their daily schedules (Bisogni et al., 2007).

### 2.5 Data management and analysis

Data from the 24hr recall interviews were transferred to SPSS version 21. A dictionary of variables was prepared with all variables. 24hr recall data were then cleaned by checking for missing values and inconsistency in the data and personal information was also removed. Analysis was undertaken at an individual person level (frequency of eating for individuals; age categories, socio-economic status); and at the eating episode level (EDNP/EDNR score for the eating episode; length of eating episode; time of day of eating episode). Participants from the two cities in Ghana were merged for the analysis because we were interested in eating practices at country, rather than, city level. Descriptive statistics were calculated and visualised to explore practices in Ghana and Kenya. Negative binomial regression models were used to analyse the influence of SES on count of food types consumed for individuals. Statistical analyses were completed using R.

## 3 Results

We slightly over-recruited based on our target quota sample, with a total of n = 459 participants (female/and male, adolescents/adults aged ≥13 y) across both countries: Ghana (n = 198 Accra, *n* = 103 Ho) and Kenya (*n* = 158). See Table 3.

**Table 3:** Socio-demographic characteristics of the sample.

	Total (*n* = 459)		Accra (*n* = 198)		Ho (*n* = 103)		Nairobi (*n* = 158)	
	*n*	%	*n*	%	*n*	%	*n*	%
**Gender**								
*Females*	310	67.5	122	61.6	103	100.0	85	53.8
*Males*	149	32.5	76	38.4	_	_	73	46.2
**Age**								
*13-18y*	150	32.7	65	32.8	37	35.9	48	30.4
*19-49y*	205	44.7	83	41.9	66	64.1	56	35.4
*≥ 50y*	104	22.6	50	25.3	_	_	54	34.2
**Socio-economic status**^a^								
*Lowest*	222	48.4	97	49.0	51	49.5	74	46.8
*Low to middle*	237	51.6	101	51.0	52	50.5	84	53.2
**Occupation**								
*In work*	193	42.0	74	37.4	37	35.9	82	51.9
*In education*	76	16.6	28	14.1	16	15.5	32	20.3
*Not in work or education*	190	41.4	96	48.5	50	48.6	44	27.8
**Body mass index**								
*<*25 kg/m^2^	225	49.1	99	50.2	47	45.6	79	50.0
*≥*25 kg/m^2^	233	50.9	98	49.8	56	54.4	79	50.0

a Participants were selected if they were classified as: ‘lowest SES’ (1st quintile); ‘low to middle SES’ (2nd and 3rd quintiles).

### 3.1 Food group consumption

**Fig. 1:**
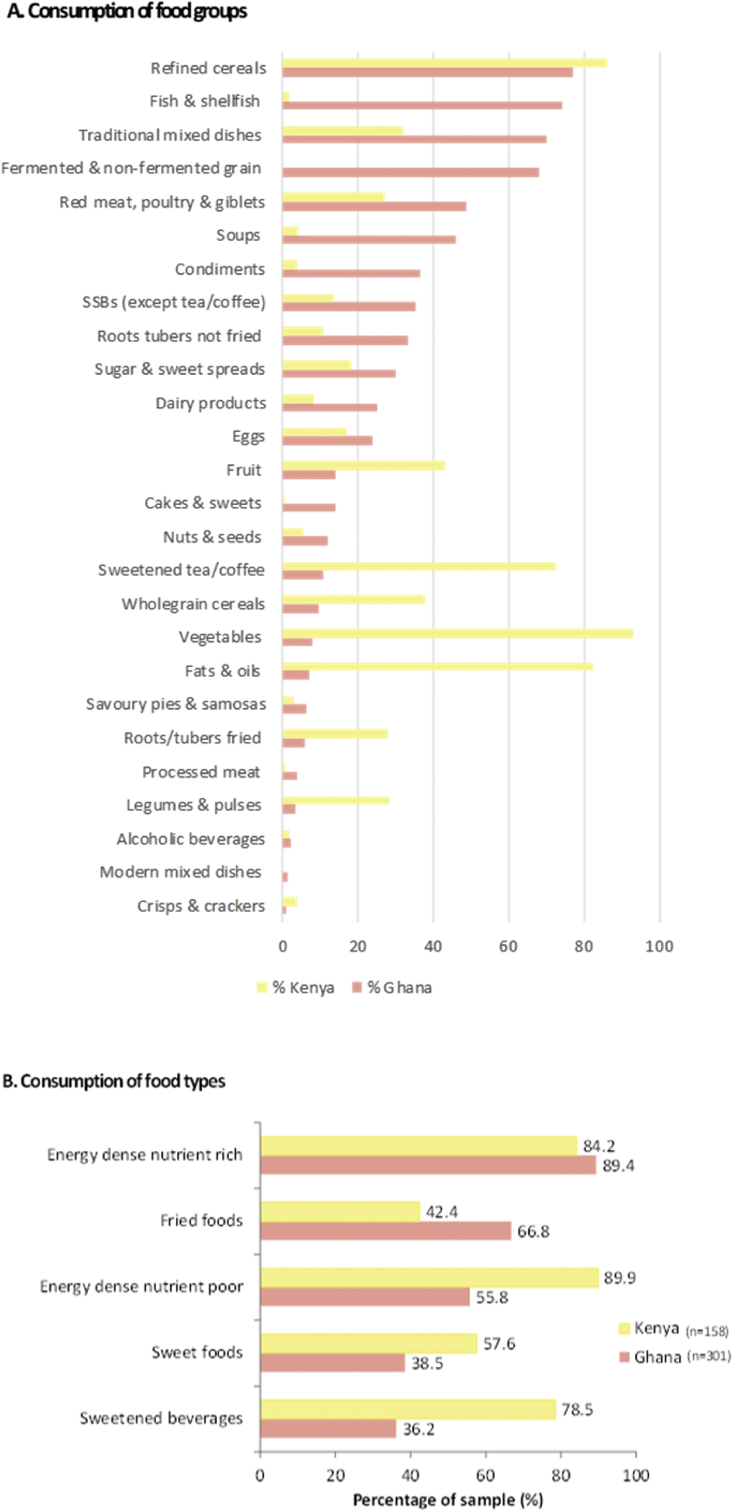
Consumption of food groups (A) and unhealthy foods (B).

A list of all food items consumed from the 24hr recall yielded a total of *n* = 138 unique foods for Ghana and *n* = 136 for Kenya (Table 1). In terms of overall food consumption, we found evidence that nutrition transition existed in both countries when compared with theories of food consumption in the context of nutrition transition, but in slightly different ways (Fig. 1-A). There was widespread consumption of vegetable oils in Kenya (82.3%), refined cereals in both countries (77.1% Ghana; 86.1% Kenya), but lower consumption of unrefined wholegrain cereals (9.6% Ghana; 38.0% Kenya). There was widespread consumption of animal source foods, including fish in Ghana (74.4% Ghana; but only 1.9% Kenya) and red meat and poultry (especially in Ghana, with 48.8% of the sample consuming them on the previous day, compared with 27.2% in Kenya). Eggs and dairy product consumption was less widespread (Fig. 1-A). Fruit and vegetable consumption was higher in Kenya (43.0% and 93.0% respectively), compared to Ghana (14.3% and 8.0% respectively) (Fig. 1-A). Consumption of legumes/pulses was higher in Kenya than in Ghana, but in Ghana this did not account for traditional mixed dishes, stews or soups that contained beans/pulses. There was widespread consumption of SSBs, including sweetened tea/coffee in Kenya (72.8% of Kenyans consuming, compared with 11.0% of Ghanaians) and SSBs (excluding tea/coffee) in Ghana (35.2% of Ghanaians compared with 13.9% of Kenyans). However, highly processed food group consumption (processed meats, crisps and crackers) was low in both countries. Overall, consumption of so called ‘ultra-processed’ food consumption was low in these deprived neighbourhoods in both countries and was restricted to consumption of noodles, fried sausage, corned beef, jam, ketchup, tomato paste, SSBs, sweetened milk, cocoa milk drinks and confectionary.

Fig. 1 here Consumption of food groups and unhealthy foods.

### 3.2 ‘Unhealthy’ food consumption

Consumption of food and beverages classified as EDNP (e.g. biscuits, doughnuts, meat pie, fried sausage, sweets and toffee, oils and fats - Table 1) was widespread, especially in Kenya where 89.9% of eating episodes contained EDNP items, compared with 55.8% of eating episodes in Ghana (Fig. 1-B). EDNR foods and beverages (e.g. peanut butter, plantain, waakye-rice and beans, boiled red meat, mabuyu-baobab fruit candy- Table 1) were even more widespread, with the majority of participants in each county consuming these the previous day (89.4% Ghana, 84.2% Kenya). In terms of unhealthy food types, over a third of participants in Ghana (38.5%) compared with over half in Kenya (57.6%) ate sweet foods in the 24hr period before the interview. SSBs were consumed at three-quarters of eating episodes in Kenya (78.5%) and over a third in Ghana (36.2%), with those in Kenya coming primarily from sweet tea/coffee. Two-thirds of eating episodes (66.8%) in Kenya contained fried foods, compared with 42.4% in Ghana.

We explored whether consumption of these categories of foods (EDNP, EDNR, sweet foods, SSBs or fried foods) were more or less common in low or middle SES populations for each country (**Supplementary file 4: SES analysis**). Our analyses suggested that most unhealthy categories (especially sweet foods, SSBs or fried foods), were more commonly consumed in lower SES individuals. Associations were consistent in both countries. Effect sizes were larger in Kenya compared to Ghana, suggesting stronger socio-economic influences and inequalities operating in Kenya.

### 3.3 Periodicity of eating practices

**Fig. 2:**
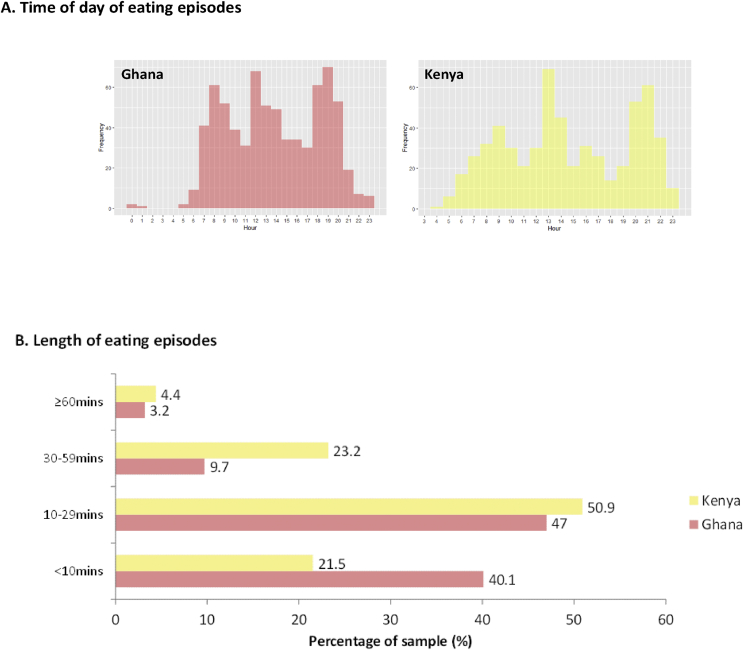
Timing (A) and length (B) of eating episodes.

A structured meal pattern around three main meals a day in both countries was evident, as participants reported eating an average of 3.3 times a day in Ghana and 3.7 times in Kenya. (Fig. 2-A). There was limited snacking in-between meals in Ghana and evidence of an afternoon snack in Kenya. The eating day started earlier in Ghana: breakfast (7–8am), lunch (12-1pm) and dinner (5–7pm), compared with Kenya: breakfast (8–9am), lunch (1–2pm) and dinner (8–9pm), with a snack more likely between lunch and dinner in Kenya. We defined breakfast, lunch and dinner time periods based on the peak times across the sample in each country.

Fig. 2 Structure and length of eating episodes.

**Fig. 3:**
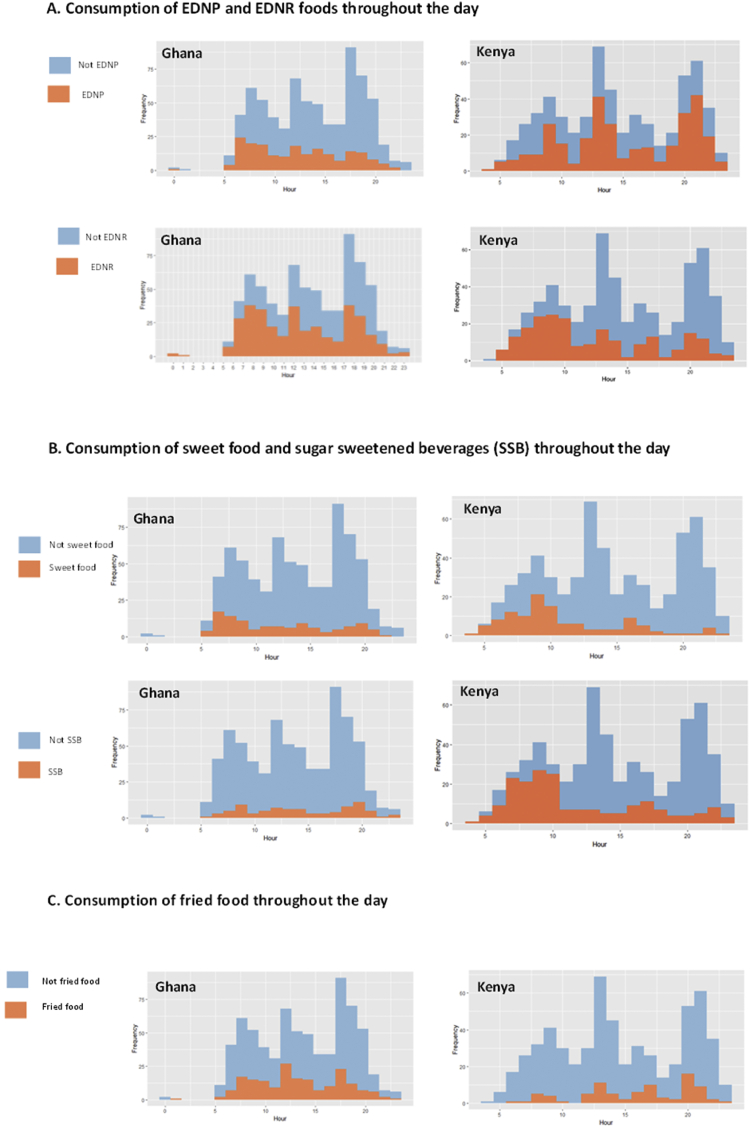
Healthiness of eating episodes throughout the day based on nutrients (A), sweet foods and beverages (B) and fried food (C).

In Ghana, EDNP foods were more commonly consumed during the morning, whereas in Kenya, EDNP food consumption peaked at meal times. EDNR food consumption peaked at meal times in Ghana. These patterns suggest that EDNP foods are a more integral part of meal times in Kenya, compared with EDNR foods in Ghana (Fig. 3-A). Consumption of sweet foods peaked in the morning in both countries (Fig. 3-B). In Ghana, SSBs consumption tended to peak with or just after mealtimes, whereas in Kenya they peaked at breakfast time and in the afternoon. In Ghana, fried food was an integral part of all mealtimes, whereas in Kenya, fried food was particularly common with the evening meal (Fig. 3-C). SSBs appear to be more common in-between meals in both countries, but there is also a high consumption of SSBs at breakfast in Kenya (Fig. 3-B), coming mainly from tea.

Fig. 3 Healthiness of eating episodes throughout the day.

### 3.4 Tempo of eating practices

In Ghana, the majority of eating episodes were either <10 min (40.1%) or 10–29 min (47.0%) (Fig. 2-B). People took longer to eat in Kenya, where less eating episodes were <10 min (21.5%). Longer eating episodes (≥30 min) were twice as likely in Kenya (27.6% vs 12.9% in Ghana). Almost one-third (31.5%) of the shortest eating episodes (<10 min) included EDNP foods in Ghana, compared with almost half (49.2%) in Kenya. In Ghana, sweetened beverages were more likely to be consumed at shorter eating episodes (17.4% of episodes <10 min v 6% of episodes ≥30 min). The opposite trend was apparent in Kenya (29.4% v 39.5% respectively), where SSBs (mostly from sweetened tea/coffee) were more likely to be consumed at longer eating episodes, suggesting they are integrated more into family meals. Longer eating episodes were more likely to have a greater intake of fried food in Ghana (19.6% v 43% respectively) and EDNP foods in Kenya (49.2% v 56.8% respectively).

### 3.5 Synchronisation of eating practices

**Fig. 4:**
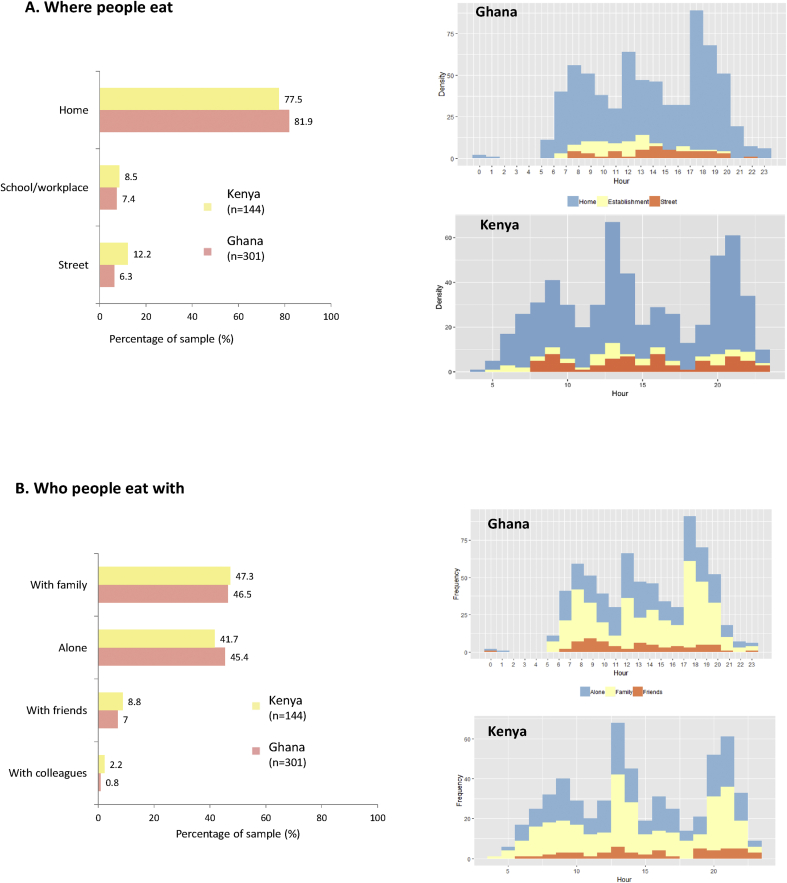
Synchronisation of eating practices incorporating where people eat (A) and with whom (B).

The home environment appeared to be a key setting for shaping food consumption (Fig. 4-A), given that over three-quarters of eating episodes were taken at home in both countries (81.9% in Ghana v77.5% in Kenya), especially the evening meal in both countries (Fig. 4-A). Longer meals were also more likely to occur at home. In both countries, street eating was not a large contributor to food habits, as only 6.3% (Ghana) and 12.2% (Kenya) of eating episodes were taken on the street; it was most common in the afternoon (Fig. 4-A). Schools and workplaces were the least common settings for food consumption in both countries (7.4–8.5% of food episodes). This low percentage may be because school feeding programmes in both countries focus on primary schools (so not on secondary schools with adolescents aged ≥13 years as in our study). The healthiness of food consumed varied across countries. In Ghana: unhealthier foods were eaten in schools/workplaces; whereas in Kenya: unhealthier foods tended to be eaten less often in schools/workplaces (less EDNP or sweetened beverages) (data not shown).

Fig. 4 Synchronisation of eating practices.

Eating with friends was much less common than with family, as only 7–8.8% of eating episodes were eaten with friends (Fig. 4-B). Fried foods were almost three times as likely to be consumed when with friends in Ghana (30.3% in Ghana v 11.5% in Kenya). This was not the case in Kenya, where sweet foods and SSBs were more commonly consumed when with friends (sweet foods 23.1% in Kenya v 15.2% in Ghana; SSBs 30.8% in Kenya v 24.2% in Ghana). Breakfast was the most frequent meal eaten with friends in Ghana, whereas the evening meal in Kenya was more likely to be shared with friends than other meals (Fig. 4-B). However, eating alone was also common in both countries, as 41.7% in Kenya and 45.4% in Ghana of eating episodes were taken alone (Fig. 4-B). Eating alone was most common at lunchtime in Kenya and in the evening in Ghana. The role of family was core, as 46.5% in Kenya and 47.3% in Ghana of eating episodes were taken with family. In Ghana, family mealtimes were less likely to include EDNP foods (24.5%) than in Kenya (53.4%).

Analysis was also undertaken for sex and age across periodicity, tempo and synchronisation, but no differences emerged (data not shown).

## 4 Discussion

This study investigated how unhealthy food and beverages are embedded in everyday life in deprived areas of two African countries (Ghana and Kenya), to identify levers for context relevant policy to prevent multiple forms of malnutrition.

### 4.1 Food consumption

We found evidence of unhealthy diets in deprived communities in both countries, which reflected the types of diets expected in countries undergoing the nutrition transition, with widespread consumption of refined cereals in both countries. Animal source foods were commonly consumed, including red meat, poultry and fish (especially in Ghana). Consumption of unhealthy food types was common, especially SSBs in Kenya and fried foods in Ghana. Consumption of EDNP food and beverages was common, especially in Kenya. Commonly consumed EDNP foods were sugar, coco milk drinks, fried chicken in Ghana and sugar, vegetable oil and margarine in Kenya. Consumption of EDNR (often traditional foods associated with cultural heritage) was widespread, with >84% of the sample consuming these in both countries. Commonly consumed EDNR foods were fried fish, white sugar/butter bread and boiled red meat in Ghana and mandazi (African doughnut), white bread and groundnuts in Kenya. Sweet foods and SSBs were popular in both countries, but an appreciation of sweetness was evident in Kenya across all eating occasions, with SSBs coming primarily from sweet tea/coffee at breakfast and in the afternoon. The Kenya STEPS survey (Kenya Bureau of Statistics, 2015) reported that over a quarter (28%) of Kenyans always add sugar to beverages. Evidence from a recent systematic review and meta-analysis of 47 studies of dietary behaviours among adults and adolescents in Ghana and Kenya also found some evidence of nutrition transition with relatively widespread consumption of animal source foods (especially red meat and poultry), unhealthy foods and beverages, and particularly SSBs, which were consumed by 39.9% of the population in Ghana/Kenya (Rousham et al., 2020).

Consumption of so called ‘ultra-processed’ food and beverages (Monteiro et al., 2010) was low in the deprived neighbourhoods studied in both countries. Ultra-processed foods are energy dense and characterized by high levels of free sugar, total/saturated/trans fats, sodium and low levels of protein and fibre (Monteiro et al., 2010; Moubarac et al., 2013). ‘Ultra-processed’ food and beverages overlap to some extent with the classification of EDNP foods and beverages that we used. But it was regarded as less appropriate for our context as ultra-processed food does not account for the presence of other beneficial nutrients to include in the diet, besides fibre. A strength of our approach to categorising foods was that it is based on previously validated approaches (e.g. Drewnowski and Fulgoni, 2014) and by including several nutrients to encourage, it accounts for the context of multiple burdens of malnutrition. Therefore, taking this approach means that identifying subsequent interventions can emphasize foods to encourage as well as to avoid, hence shifting the notion of a ‘healthy’ food-based on the absence of fat, sugars and sodium to also encompass its content of beneficial nutrients (Drewnowski, 2005). Low SES groups were more likely to consume unhealthy foods. It has been postulated that in times of economic stress, low SES groups tend to choose cheaper energy-dense foods to maximize energy value for money, resulting in habitual energy-dense, nutrient-poor diets (Drewnowski and Specter 2004). Our findings suggest a similar SES gradient to that of high income countries, i.e. low SES are more likely to consume an unhealthy diet (Allen et al., 2017). A limitation with using a qualitative 24h recall was that we did not have data on portion size, which also contributes to energy intake and therefore needs acknowledging in future interventions and policy. The use of quota sampling allowed us to include a diverse socio-demographic background in the selected deprived communities, but a larger quantitative study would have allowed us to explore differences in food consumption within population subgroups, but this was not the purpose of this study.

There are several limitations with the method used for classifying foods as unhealthy. The classification of some foods as EDNP was counter-intuitive and may be a result of the widely used 10% cut off we used, but it could lead to the classification of some foods as EDNP or EDNR when they are not, and further validation is required. For example, in Ghana, meat pies were classified as EDNP because there is a high ratio of pastry to filling in meat pies, so nutrient density is less than one may expect from other contexts. This is the same logic behind other fried meats/sausages. We acknowledge that these foods have positive nutrients (protein, iron, zinc) but the negative nutrients outweigh them (either because the overall score for negative nutrients is higher or very close). Fried chicken was classified as nutrient poor in Ghana because the nutrient values suggested a higher fat and lower micronutrient content than the food composition tables used in Kenya, where it was classified as nutrient rich. This emphasises the challenge of using different food composition tables, as well as the diversity in nutrient composition of some foods depending on context. Indeed, this potential misclassification of foods seems to be a limitation of nutrient only approaches to classifying foods and beverages, as the classification is dependent on the accuracy of the food composition foods in the context where they are used. Not all foods and beverages consumed were listed in one food composition table for Ghana or Kenya and we had to consult other food composition tables to complete these for missing foods or nutrients (**outlined in Supplementary File 3**).

### 4.2 Eating practices

A structured meal pattern around three main meals a day in both countries was evident with limited snacking in-between meals, except in the afternoon in Kenya. These findings are in line with evidence from a systematic review (Rousham et al., 2020), in which most individuals and households had a typical pattern of three meals per day in Ghana and Kenya. The greater likelihood of a regular afternoon energy dense snack (usually sweetened tea, with chapatti or mandazi-doughnut in Kenya) appears to be a reflection of the later timing of the evening meal compared with Ghana. We need to acknowledge this is part of eating practices and encourage healthy foods/less sugar at those times through FBDGs and subsidies on healthier options, like fruit. EDNP foods were a more integral part of meal times in Kenya, whereas EDNR and fried food were well integrated into Ghanaian meals. Indeed, the adult Ghanaian diet is traditionally energy-dense; the main energy-dense component (grain, cereal, legume or tuber) of the diet is served with soup or stew, usually accompanied by fish, beef or poultry (Laar and Aryeetey, 2014), which is characteristic of cultural eating practices of many sub-Saharan African countries. By ‘traditional’ we draw on a range of definitions of traditional food that usually evoke cultural or gastronomic heritage, sharing of knowledge, usually within a country/region (Sebastia, 2017). In one study of Ghanaians, participants referred to traditional foods as commonly eaten, culturally acceptable foods associated with national cultural identity (Osei-Kwasi et al., 2019-b). Defining ‘traditional’ food and diets is challenging given that colonisation in both countries by the British has incorporated foods that have been part of diets for more than a generation. For example, in Ghana, people in the urban south, where the British predominantly resided, incorporated milk, tea and breakfast cereals in their regular diet (Tuomainen, 2009), which illustrates the challenge of defining ‘traditional’.

Most eating episodes were relatively short, as around three-quarters were <30 min, with people taking longer to eat in Kenya. We have not identified any similar studies in Africa, but we know from the UK and European context that the length of time spent eating varies across cultures. One UK study estimated that over three-quarters (79–83%) of meals lasted 10–30 min and almost a quarter (17–21%) were longer, lasting ≥30 min, which is similar to the Ghanaian and Kenyan context (Cheng et al., 2007). A European study reported that the French spend almost 3 h/day eating whilst the Finnish, Slovenian, Estonian and British spend <2 h/day (Warde et al., 2007). They found that time spent eating has reduced over the previous decades, suggesting we might expect the same in countries undergoing transition. As we did not collect data on the exact number of minutes per episode but as a time category, we are unable to make direct comparisons with these other studies. Nevertheless, given the distribution of time spent eating, we can reasonably conclude that in Ghana and Kenya, it is less than in these European countries.

Almost one-third of the quickest eating episodes (<10 min) included EDNP foods in Ghana, compared with almost half in Kenya. In Ghana, sweetened beverages were more likely to be consumed at shorter eating episodes, but the opposite trend was apparent in Kenya, where SSBs tended to be consumed at longer eating episodes, suggesting they are integrated more into family meals. In both countries, longer eating episodes were more likely to have a greater intake of EDNR foods, fried food in Ghana and EDNP foods in Kenya. Other studies have reported that short durations of eating have been attributed to consuming so called ‘fast foods’ and more snacking and individualized eating (Southerton 2011). Eating alone was common in both countries, involving over a third of eating episodes. Nevertheless, street eating was not a large contributor to food consumption, even though it was twice as likely in Kenya. One limitation from our study is that we are unable to identify where food eaten at home was prepared, and it is possible that food may have been purchased from street vendors but consumed at home, possibly because work in these deprived communities is more likely to be informal and close to home. Policy and interventions, including FBDGs, need to recognise that quick and convenient options are required that are also healthy and can be eaten alone.

The home environment and the family emerged as an important setting where healthier eating can be encouraged, with more than three-quarters of meals consumed at home and almost half of eating episodes taken with family, which tended to be energy dense and fried/high in sugar. Eating with friends was much less common than with family. This was also the case in a US study (Sobal and Nelson, 2003), where authors report that commensal relationships are primarily with family. Eating alone was most common at lunchtime in Kenya and in the evening in Ghana. This did not follow the trend we had expected from studies in high income countries (Sobal and Nelson, 2003), where people tend to eat alone more in the day but share evening meals with family. Whilst eating with families peaked in the evening in Ghana, so did eating alone. One explanation may be that people's working lives are less structured in Ghana and Kenya, so families may not gather together as much in the evening due to irregular work patterns; possibly because family members may arrive home late after work when others have already eaten. Eating routines tend to be embedded in work and family schedules (Jastran et al., 2009; Warde, 2015), but one limitation of our study is that we did not measure working patterns so we are unable to shed light on their inter-relation with food consumption and eating practices in these deprived communities in Ghana and Kenya. A strength of our study was the inclusion of situational information on eating practices integrated within the qualitative 24hr recall that we have additionally linked to the healthiness of eating episodes. Most studies of dietary intake only focus on food/beverage consumption, rather than also investigating the eating practices around them (Sobal et al., 2012).

Whilst we are unable to generalise to a wider urban population in both countries, our purpose was to undertake an in-depth investigation in low income populations who suffer most from multiple burdens of malnutrition, whilst policy action often ignores SES and is insufficiently sensitive to the daily lives of the urban poor, but tends to be targeted at the whole population.

## 5 Recommendations for policy action and conclusions

In the deprived urban neighbourhoods studied in Ghana and Kenya, we found widespread consumption of unhealthy foods and beverages, with high consumption of EDNP, EDNR foods and fried/sweet foods. Our findings have provided evidence for action in the following three policy areas:

*1.Enhancing financial and physical access to healthier foods that are convenient (can be eaten quickly/alone) through for example, subsidies and incentives/training for local food vendors*.

We make this recommendation based on our findings that food episodes are often relatively quick so they need to be healthy and convenient. Local food vendors are omnipresent in the neighbourhood food environment, as demonstrated by our geographical mapping study on the physical food environment in these same neighbourhoods (Green et al., 2020), so they could also play a key role in providing healthy food. We think that this wider context needs to be acknowledged in the policy actions we are recommending. We mention financial access because these are deprived areas, and we know from many studies in Africa, including our community participatory studies in these same neighbourhoods (Pradeilles et al., 2019-b) that the cost of food is a major driver of food choice. Indeed we found that sweet foods, SSBs or fried foods were more commonly consumed amongst the lowest SES categories in our study.

*2.* *Actions to limit access to unhealthy foods and beverages through, for example, fiscal and advertising policies to dis-incentivise unhealthy food consumption, and 'processed' SSBs, especially in Ghana.*

We make this recommendation because we found that 'processed' SSBs consumption (soft/fruit drinks, sodas and sweetened beverages, sweetened milk drinks) was widespread, especially in Ghana. We know from evidence in these same deprived neighbourhoods (Green et al., 2020) that advertising of SSBs (except tea/coffee) is widespread, comprising almost half of all advertisements. We also know from wider research that advertising is an important driver of food choice. We also know from policy appraisals with national stakeholders in Ghana and Kenya that food advertising controls are a priority for action (Laar et al., 2020; Asiki et al., 2020). We recommend actions to dis-incentivise unhealthy food consumption because we found widespread consumption of unhealthy foods and beverages, including at mealtimes. Limiting access to these foods by making healthier foods (such as fruit and vegetables) relatively cheaper (through subsidies on healthier foods or taxes on unhealthy food and beverages) could contribute to a healthier food environment.

*3.* *Introducing or adapting FBDGs incorporating advice on reducing sugar and fat at mealtimes accompanied by cooking skills interventions focussing on reducing frying/oil used when preparing meals, including ‘traditional’ foods/dishes and reducing the sugar content of breakfast (from foods and drinks)*.

We found that sweetened beverages were consumed at three-quarters of eating episodes in Kenya (78.5%) and over a third in Ghana (36.2%), with those in Kenya coming primarily from sweet tea/coffee. We also found that EDNP foods and fried foods were an integral part of meal times in Kenya, compared with EDNR and fried foods in Ghana. Consumption of sweet foods and SSBs peaked at breakfast in Kenya (just sweet foods increase at breakfast in Ghana). When snacking occurred (more common in Kenya), it was in the afternoon and tended to be accompanied by a sweetened drink. In both countries, fried food was an integral part of all mealtimes, particularly common with the evening meal in Kenya. SSBs appear to be more common in-between meals in both countries, but there is also a high consumption of sweetened drinks at breakfast in Kenya, coming mainly from tea. Therefore, FBDGs need to acknowledge these directly, for example, making recommendations for a lower sugar breakfast by reducing sugar in tea in Kenya and sweet food consumption in both countries; and reducing fat in meals, including those consumed at home, including ‘traditional’ foods and dishes that are associated with cultural identity. In Ghana there are no interpretive, evidence-informed FBDGs despite political support (Laar et al., 2020). However, Dietary and Physical Activity Guidelines have been adopted by the Ghana Dietetic Association (MoH 2009), which provide information on making healthy choices and planning meals based on the nutrient content of foods. Kenya has published national Guidelines for Healthy Diets and Physical Activity that provide generic guidance (MoH, 2017). Both of these guidelines are nutrient focused and do not frame messages in terms of how or when food is eaten as part of meals. They do not mention avoiding sweet foods/beverages (at breakfast), fried foods or SSBs. They also need to account for who people eat with-family meals or alone and include examples of healthy convenient foods. All this could be added to extend their reach, including to more deprived communities.

## Funding

This work was supported by two funders in two research projects. The ‘Dietary transitions in Ghana’ project was funded by a grant from the Drivers of Food Choice Competitive Grants Programme [grant number OPP1110043], which is funded by the 10.13039/100000865Bill & Melinda Gates Foundation and 10.13039/501100002992The Foreign, Commonwealth and Development Office and managed by the 10.13039/100008899University of South Carolina Arnold School of Public Health, USA. The TACLED project was funded by a Global Challenges Research Fund Foundation Award led by the 10.13039/501100000265MRC [grant number MR/P025153/1], and supported by 10.13039/501100000267AHRC, 10.13039/501100000268BBSRC, 10.13039/501100000269ESRC and NERC. The funders played no role in the design of the study, data collection, data analysis, interpretation of the data or writing of the publication.

## Declaration of competing interest

The authors declare that they have no known competing financial interests or personal relationships that could have influenced the work reported in this paper.
