# Supplementary material for: Unhealthy eating practices of city-dwelling Africans in deprived neighbourhoods: Evidence for policy action from Ghana and Kenya
Source: Glob Food Sec. 2020 Sep;26:100452. doi: 10.1016/j.gfs.2020.100452 (PMC7726234; doi:10.1016/j.gfs.2020.100452)
Supplement: Multimedia component 1 [file mmc1.docx]

Supplementary file 1- Quota sampling

| **Table 1a: Quota sampling plan in Accra (DFC project)** | | | | | | | | | |
| --- | --- | --- | --- | --- | --- | --- | --- | --- | --- |
| **SES**  **Reproductive**  **Life Course** | Lowest SES | | | | Low to middle SES | | | | **N** |
| 13-14y (not pregnant or  lactating) | Not in work or education | | In work or education | | Not in work or education | | In work or education | | 24♀ |
|  | BMI<25 | BMI≥25 | BMI<25 | BMI≥25 | BMI<25 | BMI≥25 | BMI<25 | BMI≥25 |  |
| 15-49y (not pregnant or lactating) | Not in work or education | | In work or education | | Not in work or education | | In work or education | | 24♀ |
|  | BMI<25 | BMI≥25 | BMI<25 | BMI≥25 | BMI<25 | BMI≥25 | BMI<25 | BMI≥25 |  |
| 15-49y (pregnant) | Not in work or education | | In work or education | | Not in work or education | | In work or education | | 24♀ |
|  | BMI<25 | BMI≥25 | BMI<25 | BMI≥25 | BMI<25 | BMI≥25 | BMI<25 | BMI≥25 |  |
| 15-49y (lactating) | Not in work or education | | In work or education | | Not in work or education | | In work or education | | 24♀ |
|  | BMI<25 | BMI≥25 | BMI<25 | BMI≥25 | BMI<25 | BMI≥25 | BMI<25 | BMI≥25 |  |
| 3 participants per cell | | | | | | | | | |
| **Total sample females** | | | | | | | | | **n=96** |

**Table 1b: Quota sampling plan for in Ho (DFC project)**

|  | | | | | | | | | |
| --- | --- | --- | --- | --- | --- | --- | --- | --- | --- |
| **SES**  **Reproductive**  **Life Course** | Lowest SES | | | | Low to middle SES | | | | **N** |
| 13-14y (not pregnant or  lactating) | Not in work or education | | In work or education | | Not in work or education | | In work or education | | 24 ♀ |
|  | BMI<25 | BMI≥25 | BMI<25 | BMI≥25 | BMI<25 | BMI≥25 | BMI<25 | BMI≥25 |  |
| 15-49y (not pregnant or lactating) | Not in work or education | | In work or education | | Not in work or education | | In work or education | | 24 ♀ |
|  | BMI<25 | BMI≥25 | BMI<25 | BMI≥25 | BMI<25 | BMI≥25 | BMI<25 | BMI≥25 |  |
| 15-49y (pregnant) | Not in work or education | | In work or education | | Not in work or education | | In work or education | | 24 ♀ |
|  | BMI<25 | BMI≥25 | BMI<25 | BMI≥25 | BMI<25 | BMI≥25 | BMI<25 | BMI≥25 |  |
| 15-49y (lactating) | Not in work or education | | In work or education | | Not in work or education | | In work or education | | 24 ♀ |
|  | BMI<25 | BMI≥25 | BMI<25 | BMI≥25 | BMI<25 | BMI≥25 | BMI<25 | BMI≥25 |  |
| 3 participants per cell | | | | | | | | | |
| **Total sample females** | | | | | | | | | **n=96** |

**Table 1c: Quota sampling plan in Accra (TACLED project)**

|  | | | | | | | | | |
| --- | --- | --- | --- | --- | --- | --- | --- | --- | --- |
| SES  age group | Lowest SES | | | | Low to middle SES | | | | **N** |
| 13-18y | Not in work or education | | In work or education | | Not in work or education | | In work or education | | 24 ♂ |
|  | BMI<25 | BMI≥25 | BMI<25 | BMI≥25 | BMI<25 | BMI≥25 | BMI<25 | BMI≥25 |  |
| 19-49y | Not in work or education | | In work or education | | Not in work or education | | In work or education | | 24 ♂ |
|  | BMI<25 | BMI≥25 | BMI<25 | BMI≥25 | BMI<25 | BMI≥25 | BMI<25 | BMI≥25 |  |
| ≥50y | Not in work or education | | In work or education | | Not in work or education | | In work or education | | 24 ♂  24 ♀ |
|  | BMI<25 | BMI≥25 | BMI<25 | BMI≥25 | BMI<25 | BMI≥25 | BMI<25 | BMI≥25 |  |
| 3 participants per cell | | | | | | | | | |
| **Total sample** (n=24 females; 72 males) | | | | | | | | | **n=96** |

**Table 1d: Quota sampling plan for in Nairobi (TACLED project)**

|  | | | | | | | | | |
| --- | --- | --- | --- | --- | --- | --- | --- | --- | --- |
| SES  age group | Lowest SES | | | | Low to middle SES | | | | **N** |
| 13-18y | Not in work or education | | In work or education | | Not in work or education | | In work or education | | 24 ♂  24 ♀ |
|  | BMI<25 | BMI≥25 | BMI<25 | BMI≥25 | BMI<25 | BMI≥25 | BMI<25 | BMI≥25 |  |
| 19-49y | Not in work or education | | In work or education | | Not in work or education | | In work or education | | 24 ♂  24 ♀ |
|  | BMI<25 | BMI≥25 | BMI<25 | BMI≥25 | BMI<25 | BMI≥25 | BMI<25 | BMI≥25 |  |
| ≥50y | Not in work or education | | In work or education | | Not in work or education | | In work or education | | 24 ♂  24 ♀ |
|  | BMI<25 | BMI≥25 | BMI<25 | BMI≥25 | BMI<25 | BMI≥25 | BMI<25 | BMI≥25 |  |
| 3 participants per cell | | | | | | | | | |
| **Total sample** (n=72 females; 72 males) | | | | | | | | | **n=144** |

**Supplementary File 2. An example of the qualitative 24hr recall data**

|  | | | | | | | |
| --- | --- | --- | --- | --- | --- | --- | --- |
| **4a.Time of day**  Exact time as much as possible, e.g. 7.10am | **4b.Food item/dish consumed**  Add a suitable label for description, e.g. plantain, fufuo ne nkatenkwan) | **4c. Food item consumed** (allocate a code from the list below) | **4d. Ingredients of mixed dishes listed in 4b which are not single food items**  *Please list all ingredients in mixed dishes.* | **Codes for 4d** | **4e.Place where consumed**  01 = Own home  02 = Relative or friend’s home  03 = Market  04= Work  05= School  06- University  07= Sit down restaurant  08 Fast food outlet  09 In the street  96 = Other | **4f. Who consumed with**  01 = On own  02 = Relative  03 = Friend  04= Work colleagues  96 = Other (Specify) | **4g. How long the eating episode** (e.g. breakfast/in-between meal snack/lunch/dinner) lasted (in minutes)  01 = less than 10 minutes  02 = 10-29 minutes  03 = 30-59 minutes  04= 1 hour or more,  05= Other (specify) |
| 19.00pm | Mashed boiled yam | **\|_1_\|\|_1_\|\|_7_\|** |  | **\|__\|\|__\|\|__\|** | **\|_0_\|\|_1_\|**  If other _______ | **\|_0_\|\|_1_\|**  If other ___ | **\|_0_\|\|_3_\|**  If other _______ |
|  | Boiled egg | **\|_1_\|\|_2_\|\|_4_\|** |  | **\|__\|\|__\|\|__\|** |  |  |  |
|  | Tomato sauce | **\|_1_\|\|_2_\|\|_2_\|** |  | **\|__\|\|__\|\|__\|** |  |  |  |
|  | Palm oil | **\|_1_\|\|_4_\|\|_0_\|** |  | **\|__\|\|__\|\|__\|** |  |  |  |
|  | Water | **\|_1_\|\|_3_\|\|_5_\|** |  | **\|__\|\|__\|\|__\|** |  |  |  |

**Supplementary File 3: food composition tables used and nutrient profiling method**

| Ghana | To obtain food composition data for food items from the Ghana dataset:   1. The primary FCT used was the **2012 West African Food Composition Table** (WAFCT). 2. If a food item was not found in this FCT, or the nutritional information for that food item was incomplete, **the updated 2016 West African Food Composition Table** (WAFCT) was used to either obtain the full nutritional information for the food item or supplement what information was found in the 2012 WAFCT. 3. If a food item was unavailable in either of the two WAFCTs, the **2008 Tanzania Food Composition Table** (TFCT) was used. 4. In the event that the TFCT did not have any information on that food item, the **2018 Kenya Food Composition Table** (KFCT) was used. 5. If the food item was not in any of these four FCTs, the 7^th^ Edition of **McCance Widdowson UK Food Composition Table** (UFCT) was used. 6. A 6^th^ database, the **Ghana RIING database**, local to Ghana, was only consulted if a food item could not be located in any of the five earlier-mentioned FCTs. This was particularly relevant for mixed dishes that were specific to Ghana.   In Ghana, most nutrient values for total sugar were sourced from McCance & Widdowson Food Composition Table as there was hardly any data on added or total sugar in the various African food composition tables used. |
| --- | --- |
| Kenya | In Kenya, food composition data were compiled from the following sources:   1. The primary FCT used was the **2018 Kenyan Food Composition Table** (KFCT). 2. The **Tanzanian Food Composition Table** (TFCT) was used mainly to obtain vitamin E and total sugar content as these were unavailable in the KFCT for most of the foods. For some foods, fat content was unavailable in the KFCT. In these instances, total fat content was obtained from the TFCT. 3. The TFCT was also used profile foods items that were completely missing in KFCT. 4. The 2012 **West African Food Composition Table** (WAFCT) was also used for nutrients or food items that were missing in both the KFCT and TFCT. 5. Lastly, the 7^th^ Edition of the **McCance & Widdowson Food Composition** Table was mainly used for food items or nutrients that were unavailable in either the KFCT, the TFCT or the WAFCT. |

*Food Composition Tables Used*

Nutritional content (both macro- and micro-nutrient information) for each of these unique food items was then identified using a combination of food composition tables (FCTs) (6 for Ghana and 4 for Kenya). When extracting the nutritional information, the foods in the 4 FCTs with an identical name were chosen. In the event that a food item did not exist by its exact name in any of the FCTs, the closest possible alternative to that food was used instead. For example in Kenya, nutrient information for some local/traditional dark-green leafy vegetables was not found in any of the FCTs. In such cases nutrient values for spinach from the TFCT and UFCT were used, with the assumption that their nutrient values would not significantly differ from that of spinach since they are all classified as dark -green leafy vegetables.

*Nutrient Profiling Score Used*

Nutrient profiling was conducted according to the method proposed by Drewnowski and Fulgoni (2014). In order to carry out the nutrient profiling, the NR9.3 nutrient density score was used as a starting point (Drewnowski and Fulgoni, 2008). The NRF9.3 incorporates nutrients to limit (saturated fat, added sugars, sodium) and nutrients to encourage (protein, fibre, vitamins A, C, E and iron, calcium, potassium and magnesium), therefore reflecting the nutrient density of a food. In the event that data on added sugar are not readily available, as was the case for most of our FCTs, total sugar can be used as a substitute (Sluik et al., 2015, Drewnowski, 2017), since total sugars and added sugars are correlated (Fulgoni et al., 2009). Similarly, research by Drewnowski and Fulgoni (2009) indicates that our choice of using total fat in our nutrient profiling model, instead of saturated fat, is acceptable because the total fat and saturated fat contents in foods are highly correlated (Fulgoni et al., 2009). Additionally, given their public health importance to the reproductive wellbeing of women of reproductive age in Ghana and Kenya, two additional nutrients to encourage (folate and zinc) were added to the NRF9.3 model. This resulted in the NRF11.3 model, used in this study, based on balancing public health nutrition priorities in Ghana and Kenya, and the availability of food composition data for the selected nutrients.

Testing of the NRF9.3 model against the Healthy Eating Index found that models based on 100 kcal was more useful than those based on 100g (Drewnowski and Fulgoni, 2014), hence we used this approach. For each unique food item, the nutritional information per 100kcal for the 11 nutrients to encourage (protein, fibre, calcium, zinc, potassium, magnesium, iron, folate, vitamin A, vitamin C and vitamin E) and 3 nutrients to limit (total fat, sodium and total sugars) were entered in a spreadsheet.

Then, using the USDA dietary recommendations [US FDA], the % daily value (%dv) for each of those nutrients to encourage and limit that these foods supplied was calculated per 100kcal. The daily values used were based on energy intake of 2,000kcal for adults. The recommended daily allowance was based on the US RDA (Food and Drug Authority), since that is also used in Ghana and Kenya. The energy density for each food item, measured as kcal/100 gram was also entered on the same spreadsheet.

In order to prevent some foods that met over 100%dv having an effect on the resulting nutrition rich index, capping was conducted (Drewnowski, 2017). To do this, any columns that had any value above 100 for the %dv for each of the 11 positive nutrients to encourage were identified. Following this, a new column was created right next to any column that had one or more values above 100 for %dv and labelled %dv_capped.  In these new columns, all %dv were copied and pasted from the initial column, but any values that were >100 were changed to 100. This means that in this new column of %dv_capped, the maximum possible number would be 100. At the end of this step, all the %dv per 100kcal for each of the 11 nutrients had a maximum value of 100. This capping, however, was not done for the nutrients to limit, and their %dv per 100kcal was left as is (Drewnowski, 2017).

The next step was to separately sum all the %dv and %dv_capped per 100kcal for positive nutrients to encourage and negative nutrients to limit. The final step involved generating the nutrient rich index (NRI) by subtracting the sum of the nutrients to limit from the sum of the positive nutrients to encourage.

*Categorising Foods*

The food items were initially categorised as high energy dense if their energy content was >225kcal/100grams, intermediate if it was 125-225kcal/100grams and lower in energy if the energy content was <125kcal/100gram (WCRF/AICR, 2007

However, for simplicity for the purposes of the work to be undertaken in the project using the EDNP classification (social practices and GIS analyses), it was decided to use energy density cut off points from the World Cancer Research (WCRF) food items, therefore classifying them as energy dense if their energy content was >225 kcal/100g and energy low, otherwise (WCRF/AICR), 2007). WCRF/AICR 2007 (p379) states that ‘non starchy roots and tubers, veg and fruits supply 10-100kcal and are low energy dense; cereals and pulses (60-150 kcal); and lean meats and breads, poultry and fish (100-225kcal)’. It also states that ‘the aim is to reduce the average energy density of diets towards 125 kcal per 100g’ (p379).

After generating nutrient density scores using the NRF11.3 algorithms, cut offs/thresholds were applied using the US Food and Drug Administration (FDA) cut off points for defining a healthy food as a reference. As per the FDA rules, healthy foods should contain ≥ 10% DVs per serving for at least one of the following: protein, calcium, iron, vitamin A and C and fibre (U.S. Department of Health and Human Services, 2013). Food items were categorised as nutrient rich if their NRI score was >10 and nutrient poor, otherwise. Combining the nutrient and energy density information allowed us to classify food items as: energy-dense, nutrient-poor (EDNP); energy-dense, nutrient-rich (EDNR) and lower in energy, nutrient-rich (ELNR). The EDNP category represented “unhealthy foods” whilst the ELNR category represented “healthy foods” with EDNR being the intermediate category.

*Managing Missing Food Composition Data*

In Ghana (DFC data), we had data on 138 food items (we also had a further 6 classified but not for EDNP status because they were not consumed). 9 food items had missing nutrient scores only, none had missing energy scores only, 29 food items had both missing nutrient and energy scores. In these cases, we used our judgement based on similar food items to classify the energy density and nutrient density of these foods. Four researchers discussed and agreed these classifications (RP, MG, MH and NB).

In Kenya, two food items consumed were not found in any of the FCTs and therefore, the nutrient and energy density values could not be calculated as described above. These foods were therefore classified based on the values of foods that were closest to them, with assumption that their nutrient and energy density would be almost similar. These foods included sugar molasses (sukari nguru), which is closely related to sugar and hence classified as EDNP, and flavoured baobab seeds (mabuyu) which are closely related to nuts/ seeds and therefore classified as EDNR.

**References**

Drewnowski, A. (2005). Concept of a nutritious food: toward a nutrient density
score. American Journal of Clinical Nutrition, 82:721–32

Drewnowski, A. (2010). The Nutrient Rich Foods Index helps to identify healthy, affordable foods. American Journal of Clinical Nutrition, 91, 1095S-101S.

Drewnowski, A. and Fulgoni, V. L. (2014). Nutrient density: principles and evaluation tools. American Journal of Clinical Nutrition, 99, 1223s-8s.

Drewnowski, A. (2017). Measures and metrics of sustainable diets with focus on milk, yoghurt and dairy products. Nutrition Reviews, 76(1), 21-28

Fulgoni, V. L., 3RD, Keast, D. R. and Drewnowski, A. (2009). Development and validation of the nutrient-rich foods index: a tool to measure nutritional quality of foods. Journal of Nutrition, 139, 1549-54.

U.S. Department Of Health And Human Services. (2013). A Food Labelling Guide: Guidance for Industry. Food and Drugs Administration Centre for Food Safety and Applied Nutrition

US Department of Health and Human Services. <https://www.fda.gov/downloads/food/guidanceregulation/guidancedocumentsregulatoryinformation/labelingnutrition/ucm513817.pdf> Food and Drugs Administration Centre for Food Safety and Applied Nutrition.

WCRF/AICR (2007). World Cancer Research Fund/ American Institute for Cancer Research. Food, Nutrition, Physical Activity and the Prevention of Cancer: a Global Perspective. Washington, D.C: AICR, 2007

**Supplementary File 4: consumption of foods by SES**

Results from a negative binomial regression analysis of count of consumption (by different type of food) for Ghana and Kenya.

| Outcome | Ghana | | | Kenya | | |
| --- | --- | --- | --- | --- | --- | --- |
|  | IRR | Lower CI | Upper CI | IRR | Lower CI | Upper CI |
| Energy dense nutrient poor foods | 0.37 | 0.27 | 0.51 | 0.25 | 0.20 | 0.32 |
| Energy dense nutrient rich foods | 0.42 | 0.33 | 0.54 | 0.18 | 0.13 | 0.24 |
| Energy dense foods | 0.40 | 0.33 | 0.49 | 0.22 | 0.18 | 0.26 |
| Nutrient poor foods | 0.44 | 0.34 | 0.56 | 0.27 | 0.21 | 0.34 |
| Sweet foods | 0.33 | 0.21 | 0.51 | 0.15 | 0.10 | 0.23 |
| Sugar sweetened beverages | 0.84 | 0.47 | 1.51 | 0.21 | 0.14 | 0.29 |
| Fried foods | 0.51 | 0.36 | 0.74 | 0.18 | 0.11 | 0.29 |
| Note 1: IRR = Incident Rate Ratio. CI = 95% Confidence Interval. | | | |  |  |  |
| Note 2: Estimate is for middle socioeconomic status (SES) versus low SES as reference category | | | | | | |
| Note 3: Models adjusted for: age, body mass index, city (Ghana only), count of episodes. Modelled offset: Count of food consumed. | | | | | | |
